# Supplementary material for: Topological nodal line in superfluid 3He and the Anderson theorem
Source: Nat Commun. 2023 Jul 17;14:4276. doi: 10.1038/s41467-023-39977-2 (PMC10352354; doi:10.1038/s41467-023-39977-2)
Supplement: Supplementary file 1 — Supplementary Information [file 41467_2023_39977_MOESM1_ESM.pdf]

Supplementary Information for Topological nodal line in superfluid ^{3}He and the Anderson theorem

T. Kamppinen,[1] J. Rysti,[1] M.-M. Volard,[1] G.E. Volovik,[1, 2] and V.B. Eltsov[1]

[1]*Department of Applied Physics, Aalto University, POB 15100, FI-00076 AALTO, Finland*

[2]*Landau Institute for Theoretical Physics, 142432,Chernogolovka, Russia.*

Supplementary Note 1: Dirac nodal line and its topology

Polar phase belongs to the superfluid states with the so-called equal spin pairing. This means that it can be represented as an equal mixture of two superfluids with the projections of the Cooper pair spin $S_z = +1$ and $S_z = -1$. In superfluid ^{3}He the spin-orbit interaction is very small compared to the superfluid gap and can be neglected. That is why for each of the two spin projections one has the following Bogoliubov-de Gennes Hamiltonian:

$$H = v_F(p - p_F)\tau^3 + \Delta\frac{p_z}{p_F}\tau^1 \,, \tag{1}$$

where Δ is the gap amplitude, and τ^a are the Pauli matrices in the Bogoliubov-Nambu particle-hole space. The Hamiltonian is nullified on the line $p_z = 0$, $p = p_F$. This is the Dirac nodal line, which stability is supported by topology and symmetry. The corresponding topological invariant can be written in terms of the Hamiltonian [9]:

$$N = -\frac{1}{4\pi}\oint_C dl\, \tau^2 H^{-1}\nabla_l H \,. \tag{2}$$

Here the contour C of integration is around the element of the nodal line, see Fig. 1a in the main text. This integral is invariant under deformations preserving the time reversal symmetry, due to which the Hamiltonian must anti-commute with τ^2, i.e. $\{H, \tau^2\} = 0$. For each spin projection the invariant has the value $N = 1$, which also means that the Berry phase changes by π along C.

Supplementary Note 2: T^3 dependence of the superfluid gap in the nodal-line polar phase

The gap equation for the polar phase:

$$\frac{1}{g} = \int_0^1 dx x^2 \int_0^{E_{uv}} \frac{d\xi}{\sqrt{\xi^2 + \Delta^2(T)x^2}} - 2\int_0^1 dx x^2 \int_0^\infty \frac{d\xi}{\sqrt{\xi^2 + \Delta^2(T)x^2}} \frac{1}{\exp\left(\frac{\sqrt{\xi^2+\Delta^2(T)x^2}}{T}\right) + 1} \tag{3}$$

Here g is the normalized coupling, which is not important for us, since it drops out from further equations; $\Delta(T)$ is the gap amplitude; $x = \cos\mu$ shows the dependence of the gap function on the polar angle μ; E_{uv} is the ultraviolet cut-off of the logarithmically divergent integrals, which also drops out from the further equations, where the logarithmic terms cancel each other; the $x^2 = \cos^2\mu$ in the integral over x comes from the $\cos\mu$-dependence of the p-wave interaction potential $V_{\mathbf{k},\mathbf{k}'} \propto k_z k'_z$ and the gap in the polar phase. We have from Eq. (3):

$$\int_0^1 dx x^2 \int_0^\infty d\xi \left(\frac{1}{\sqrt{\xi^2 + \Delta^2(0)x^2}} - \frac{1}{\sqrt{\xi^2 + \Delta^2(T)x^2}}\right) = \tag{4}$$

$$= -2\int_0^1 dx x^2 \int_0^\infty \frac{d\xi}{\sqrt{\xi^2 + \Delta^2(T)x^2}} \frac{1}{\exp\left(\frac{\sqrt{\xi^2+\Delta^2(T)x^2}}{T}\right) + 1} \tag{5}$$

where in Eq. (4) the cut-off dropped out because of cancellation of logarithmic terms. At low T this Eq. (4) is proportional to $\Delta^2(T) - \Delta^2(0)$, while in the Eq. (5) one can take the $T = 0$ limit:

$$\int_0^1 dx x^2 \int_0^\infty d\xi \left(\frac{1}{\sqrt{\xi^2 + \Delta^2(T)x^2}} - \frac{1}{\sqrt{\xi^2 + \Delta^2(0)x^2}}\right) = \tag{6}$$

$$= 2\int_0^1 dx x^2 \int_0^\infty \frac{d\xi}{\sqrt{\xi^2 + \Delta^2(0)x^2}} \frac{1}{\exp\left(\frac{\sqrt{\xi^2+\Delta^2(0)x^2}}{T}\right) + 1} \tag{7}$$

Expansion in $\Delta^2(0) - \Delta^2(T)$ gives

$$\frac{1}{2}(\Delta^2(0) - \Delta^2(T)) \int_0^1 dx x^4 \int_0^\infty d\xi (\xi^2 + \Delta^2(0)x^2)^{-3/2} = \tag{8}$$

$$= 2\int_0^1 dx x^2 \int_0^\infty \frac{d\xi}{\sqrt{\xi^2 + \Delta^2(0)x^2}} \frac{1}{\exp\left(\frac{\sqrt{\xi^2+\Delta^2(0)x^2}}{T}\right) + 1} \tag{9}$$

or

$$\frac{1}{2}\left(1-\frac{\Delta^2(T)}{\Delta^2(0)}\right)\int_0^1 dx x^4\int_0^\infty d\xi(\xi^2+x^2)^{-3/2} = \tag{10}$$

$$= 2\int_0^\infty dx x^2\int_0^\infty \frac{d\xi}{\sqrt{\xi^2+x^2}}\frac{1}{\exp\left(\frac{\Delta(0)}{T}\sqrt{\xi^2+x^2}\right)+1} \tag{11}$$

In Eq. (11) the integration over x has been extended to ∞, because in this equation $x^2+\xi^2 \sim T^2/\Delta(0)^2 \ll 1$. Introducing cylindrical coordinates $x = r\cos\phi$, $\xi = r\sin\phi$ in Eq. (11), one obtains:

$$\frac{1}{6}\left(1-\frac{\Delta^2(T)}{\Delta^2(0)}\right)\int_0^\infty d\xi(\xi^2+1)^{-3/2} = \tag{12}$$

$$= \frac{\pi}{2}\int_0^\infty r^2 dr\frac{1}{\exp\left(r\frac{\Delta(0)}{T}\right)+1} \tag{13}$$

$$= \frac{\pi}{2}\frac{T^3}{\Delta^3(0)}\int_0^\infty \frac{r^2 dr}{e^r+1} \tag{14}$$

The integrals in Eq. (12) and in Eq. (14) are:

$$\int_0^\infty d\xi(\xi^2+1)^{-3/2} = 1\,, \tag{15}$$

$$\int_0^\infty \frac{r^2 dr}{e^r+1} = \frac{3}{2}\zeta(3)\,, \tag{16}$$

and we obtain the universal temperature dependence of the gap at low T

$$2\left(1-\frac{\Delta(T)}{\Delta(0)}\right) = \left(1-\frac{\Delta^2(T)}{\Delta^2(0)}\right) = \frac{9\pi}{2}\zeta(3)\frac{T^3}{\Delta^3(0)}\,, \tag{17}$$

or

$$1-\frac{\Delta(T)}{\Delta(0)} = \frac{9\pi}{4}\zeta(3)\frac{T^3}{\Delta^3(0)} = a\,\frac{T^3}{T_{\rm c}^3}\,, \tag{18}$$

where

$$a = \frac{9\pi}{4}\zeta(3)\left[\frac{T_{\rm c}}{\Delta(0)}\right]^3 \approx 8.5\left[\frac{T_{\rm c}}{\Delta(0)}\right]^3\,. \tag{19}$$

Using the result $\Delta(0) = 2.46T_{\rm c}$ from the Supplementary Note 3 we find $a = 0.57$. We stress that this value is obtained for the pure polar phase without impurities. Its agreement with the measurements presented in the main text supports extension of the Anderson theorem to the polar phase with the columnar non-magnetic defects.

Supplementary Note 3: The gap amplitude at $T = 0$ vs $T_{\rm c}$

From the gap equation at $T = 0$

$$\frac{1}{g} = \int_0^1 dx x^2\int_0^{E_{\rm uv}} \frac{d\xi}{\sqrt{\xi^2+\Delta^2(T)x^2}}\tanh\left(\frac{\sqrt{\xi^2+\Delta^2(T)x^2}}{2T}\right) = \tag{20}$$

$$= \int_0^1 dx x^2\int_0^{E_{\rm uv}} \frac{d\xi}{\xi}\tanh\frac{\xi}{2T_{\rm c}} = \int_0^1 dx x^2\int_0^{E_{\rm uv}} \frac{d\xi}{\sqrt{\xi^2+\Delta^2(0)x^2}} \tag{21}$$

we obtain

$$0 = \int_0^1 dx x^2\int_0^\infty d\xi\left(\frac{1}{\xi}\tanh\frac{\xi}{2T_{\rm c}} - \frac{1}{\sqrt{\xi^2+\Delta^2(0)x^2}}\right) = \int_0^1 dx x^2\int_0^\infty d\xi\left(\frac{1}{\xi}\tanh\frac{\xi\Delta_0}{2T_{\rm c}} - \frac{1}{\sqrt{\xi^2+x^2}}\right) \tag{22}$$

Introducing the parameter $\alpha = \Delta(0)/2T_\mathrm{c}$ one finds the value of α at which the function $f(\alpha)$

$$f(\alpha) = \int_0^1 dx x^2 \int_0^\infty d\xi \left(\frac{\tanh \alpha\xi}{\xi} - \frac{1}{\sqrt{\xi^2 + x^2}} \right) \tag{23}$$

crosses zero. Solving the equation numerically, we find $\alpha = 1.23$.

Supplementary Note 4: Temperature dependence of the frequency shift

The interpretation of the frequency shift measurements is complicated by the fact that theoretical models refer to the change of the frequency shift from its zero-temperature value, which is unknown experimentally. The zero-temperature shift should be determined by *extrapolation* of the measured data. Extrapolation requires some model for the data upfront. This circular dependence of data and interpretation results in the uncertainty in the zero-temperature value of the shift being the main source of uncertainty in the values of the exponent and of the coefficient in the temperature dependence of the frequency shift and thus of the gap.

A reasonable general model for the frequency shift for a superfluid with gap nodes is $\omega(T) - \omega_\mathrm{L} = b_1 + b_2(T/T_\mathrm{c})^{b_3}$ with b_1, b_2 and b_3 being the free parameters. The fit of the measured data to this model is shown in Supplementary Fig. 2. Observations at 0.1, 4 and 7 bar pressure demonstrate nearly ideal cubic temperature behavior with b_3 lying in the interval $2.9 - 3.1$ and the coefficient $a = -b_2/(2b_1)$ being very close to the clean-limit weak-coupling value of 0.57 for 4 and 7 bars and slightly larger for 0.1 bar in accordance with stronger T_c suppression at the lowest pressure. The fitted values are also robust with respect to the range of temperatures included in the fit. The upper limit of $0.5T_\mathrm{c}$ should be safe to determine the low-temperature behavior according to the calculations in Fig. 4 of Ref. [10]. In principle, the measurements at these three pressures are sufficient to prove the main messages of the paper on the presence of the nodal line and extension of the Anderson theorem in the polar phase, as well as to demonstrate the link between T_c suppression and the a coefficient value due to disorder breaking the requirements of the theorem.

The behavior at higher pressures is nevertheless interesting and provides more data for future analysis of the interplay between topology and disorder. The fit in the whole available temperature range gives values of b_3 up to 4 at 15 bar pressure. In principle, the exponent 4 is expected in the clean-limit case for the point nodes, which for confined ^{3}He in this work means the polar-distorted A (PdA) phase. We note, however, that for the PdA phase the extension of the Anderson theorem is not supposed to work and thus the exponent to expect is not known. Also the value of b_3 changes depending on the temperature range included in the fit. Thus we interpret the observation as an indication of a phase transition from the polar phase at higher temperatures to a different phase at lower temperatures with a maximum extent of the different phase, up to about $0.3T_\mathrm{c}$, at 15 bars and the range decreasing both at lower and higher pressures, recovering the polar-phase behavior in almost whole temperature range at 29.5 bars. (Note that in an inhomogenous confined sample, different phases can coexist even in the case of the second-order phase transition and the transition becomes extended in temperature).

We check this interpretation in Supplementary Fig. 3. Here the frequency shift at temperatures $0.3T_\mathrm{c} < T < 0.5T_\mathrm{c}$ is fit with the cubic power law as established for the polar phase to find the zero-temperature extrapolated shift. After subtracting the zero-temperature shift, the residual shift is fit with the power law in the same temperature range. The fit exponent b_3' comes very close to 3, even at the intermediate pressures, which confirms that in this temperature range the polar phase dominates also at these pressures. At lower temperatures the normalized frequency shift goes above the fit line for pressures 11 bar and above, with the maximum deviation observed at 15 bar. Qualitatively, it is the direction of deviation expected on transition to the PdA phase as seen in the lower-density nafen-90 [1]. Proper identification, though, requires more measurements in particular versus magnetic field direction and tipping angle of magnetization. At 0.1 bar one can see deviation of the data below the fit line at the lowest temperatures. In principle, this is the direction expected on the transition from the polar to the polar-distorted B (PdB) phase [2]. The deviation, however, is small and may be caused by inaccuracies in the temperature calibration. Finally, we note that at temperatures about and above $0.6T_\mathrm{c}$ one finds the deviation of data above the fit line. This qualitatively agrees with calculations for the polar phase in Ref. [10].

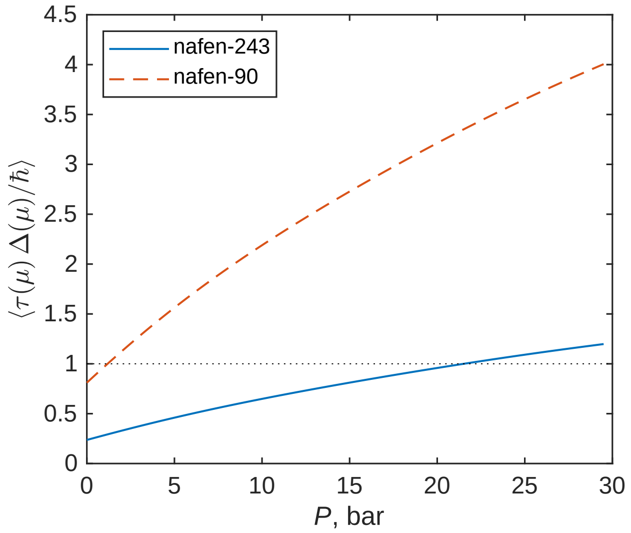

Supplementary Figure 1. Scattering in confined superfluid ^{3}He as a function of pressure. Calculated product of the quasiparticle scattering time $\tau(\mu)$ and the superfluid gap $\Delta(\mu)$ averaged over direction of the quasiparticle momentum $\mathbf{p}$ determined by the angle μ between $\mathbf{p}$ and the strands of the confining matrix. Solid line represents confining material used in this work, nafen with density 243 mg/cm^3. The dashed line is for the less dense nafen with density 90 mg/cm^3, for which phase diagram of superfluid ^{3}He states is also known [1, 2]. Here $\tau(\mu)$ is determined from $[\tau(\mu)v_\mathrm{F}]^{-1} = l_\perp^{-1}\sin\mu + l_\parallel^{-1}\cos\mu$, where v_F is the Fermi velocity and $l_\perp$ and $l_\parallel$ are quasiparticle mean free path in the direction perpendicular and parallel to the strands, respectively. For the gap, the zero-temperature value in the weak-coupling BCS theory $\Delta(\mu) = 1.23 \cdot 2k_\mathrm{B}T_\mathrm{c}\cos\mu$ is taken (see Supplementary Note 3). Thus, plotted value is an upper bound of $\tau\Delta$ as a function of temperature. For nafen-243, $\tau < \hbar/\Delta$ (except the lowest temperatures and elevated pressures), and in the major part of the phase diagram all superfluid phases are suppressed except the polar phase, which is robust due to extension of the Anderson theorem. For nafen-90, the scattering is less prominent and the polar phase is stable near T_c, while other phases replace it at lower temperatures. Values of $l_\perp$ and $l_\parallel$ are extracted from spin-diffusion measurements in the normal phase [3]. For nafen-243 $l_\perp = 70$ nm and $l_\parallel = 570$ nm while for nafen-90 $l_\perp = 290$ nm and $l_\parallel = 960$ nm.

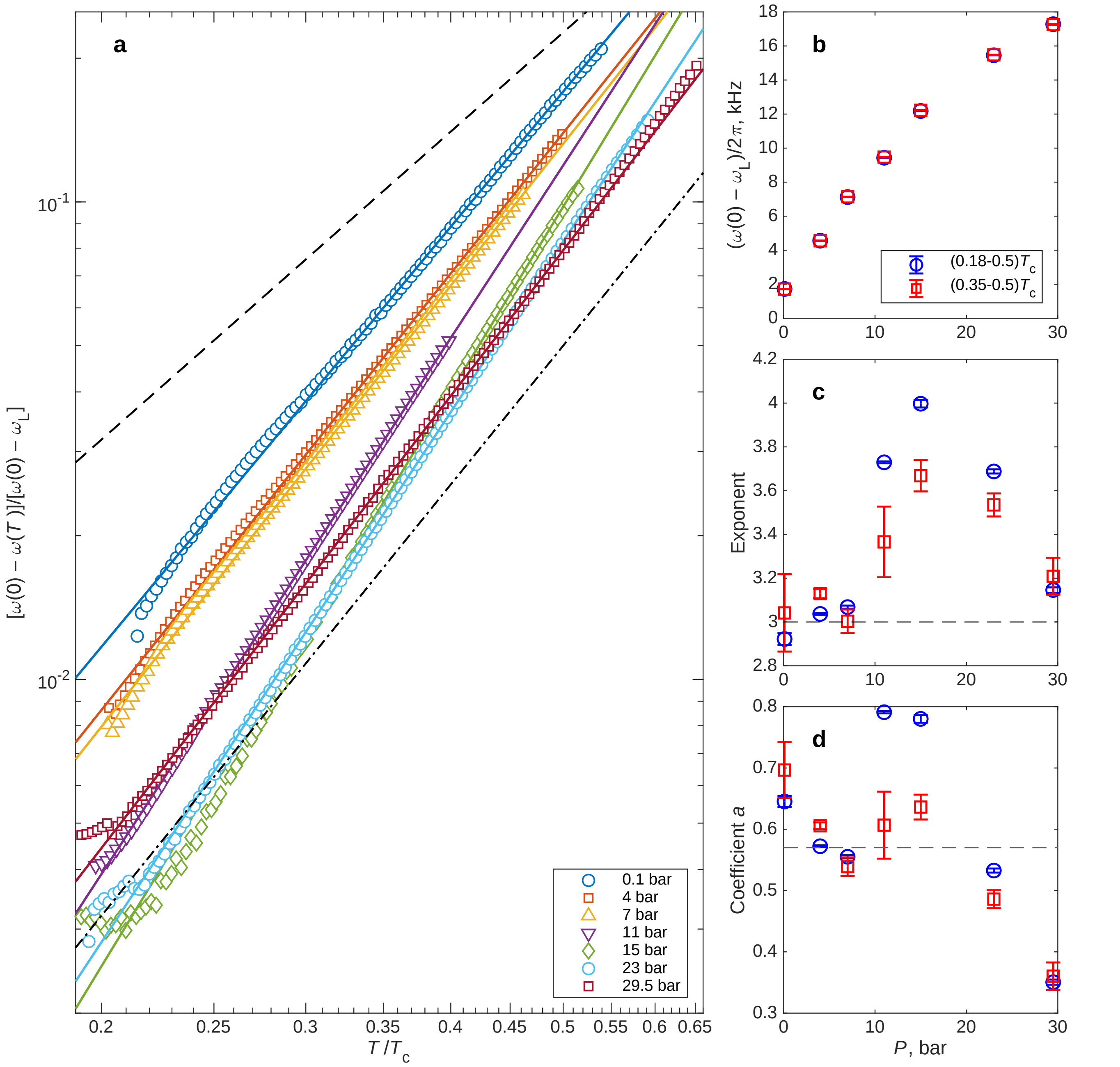

Supplementary Figure 2. Three-parameter fit of the frequency shift. The measured frequency shift of the NMR response is fitted as $\omega(T) - \omega_L = b_1 + b_2(T/T_c)^{b_3}$ with b_1, b_2 and b_3 being the fit parameters. **a**, The normalized shift, $1 - [\omega(T) - \omega_L]/b_1$ as a function of temperature at all measured pressures (symbols). The solid lines are fits performed to data at temperature $T < 0.5T_c$. Dash-dotted line corresponds to T^3 and dashed line to $T^{2.14}$. **b**, Zero-temperature shift $b_1 = \omega(0) - \omega_L$ as a function of pressure from fits to data at $T < 0.5T_c$ (circles) and at $0.35T_c < T < 0.5T_c$ (squares). Error bars show statistical uncertainties from the fit ($\pm 1\sigma$). **c**, The same plot as **b** for the exponent b_3. Dashed line shows clean-limit weak-coupling value 3 expected for the polar phase. **d**, The same plot as **b** for the coefficient $a = -b_2/(2b_1)$. Dashed line shows clean-limit weak-coupling value 0.57 expected for the polar phase. For discussion, see Supplementary Note 4.

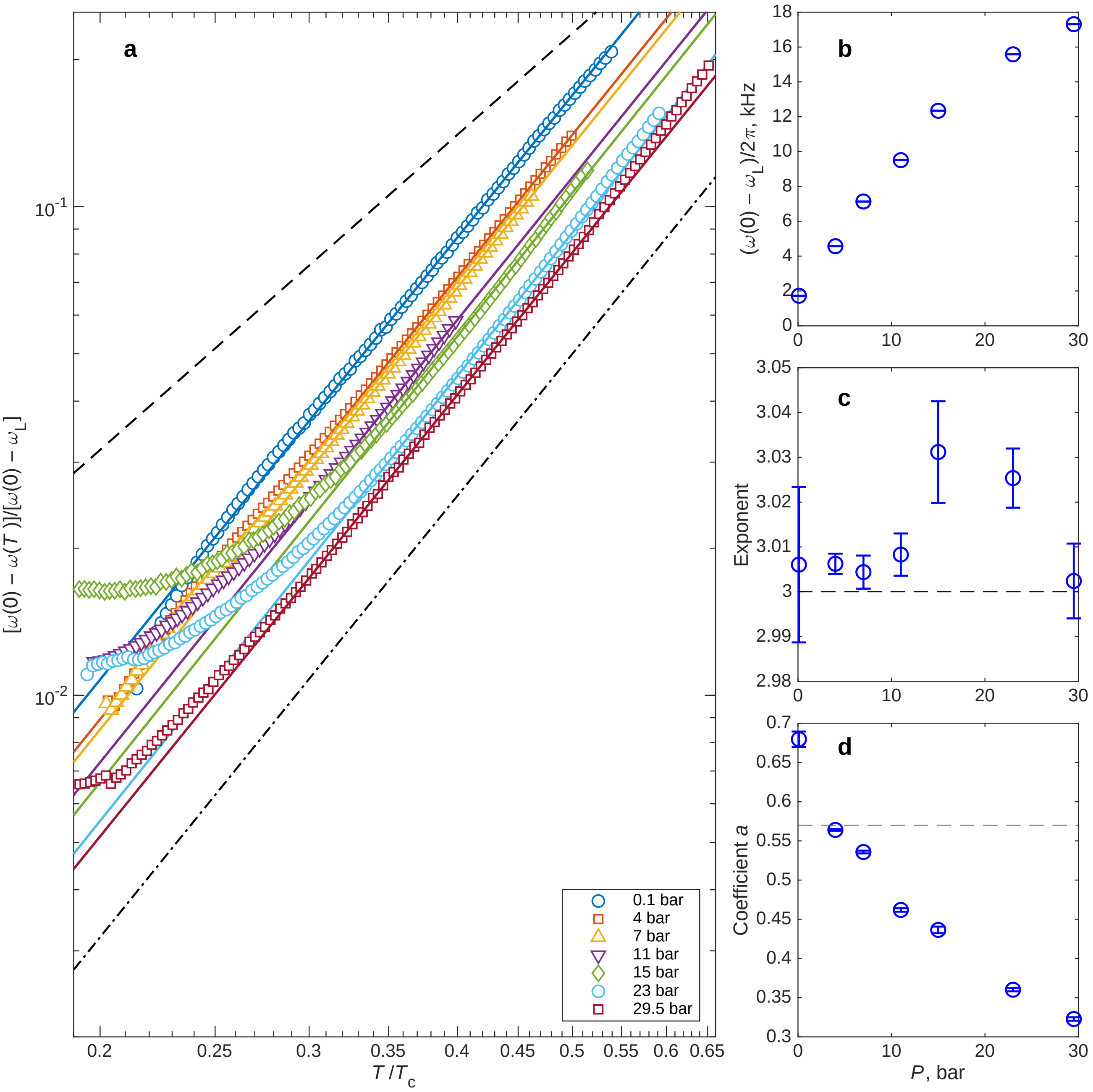

Supplementary Figure 3. Two-parameter fit of the frequency shift. The measured frequency shift of the NMR response is fitted at temperatures $0.3T_c < T < 0.5T_c$ as $\omega(T) - \omega_L = b_1 + b_2(T/T_c)^3$ with b_1 and b_2 being the fit parameters. This is the same fit as shown in Figs. 2b and 3a in the main text. **a**, The normalized shift, $1 - [\omega(T) - \omega_L]/b_1$ as a function of temperature at all measured pressures (symbols). The solid lines are fits of the normalized shift as $b_2'(T/T_c)^{b_3'}$ for data in the range $0.3T_c < T < 0.5T_c$. Dash-dotted line corresponds to T^3 and dashed line to $T^{2.14}$. **b**, Zero-temperature shift $b_1 = \omega(0) - \omega_L$ as a function of pressure. Error bars show statistical uncertainties from the fit ($\pm 1\sigma$). **c**, The same plot as **b** for the exponent b_3'. Dashed line shows clean-limit weak-coupling value 3 expected for the polar phase. **d**, The same plot as **b** for the coefficient $a = -b_2'/(2b_1)$. Within error bars, the values are indistinguishable from $-b_2/(2b_1)$ plotted in Fig. 3b in the main text. Dashed line shows clean-limit weak-coupling value 0.57 expected for the polar phase. For discussion, see Supplementary Note 4.

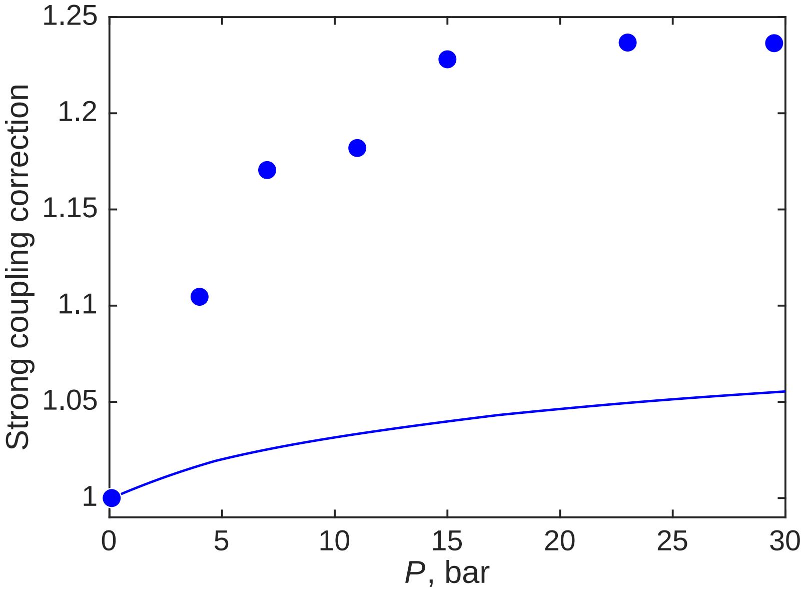

Supplementary Figure 4. Determination of the strong coupling corrections in the polar phase of ^{3}He from the frequency shift. Ratio $[\Delta(T=0,P)/T_c(P)]/[\Delta(T=0,P=0.1\,\mathrm{bar})/T_c(P=0.1\,\mathrm{bar})]$ as a function of pressure P as determined from the zero-temperature frequency shift, Fig. 4 in the main text and Supplementary Fig. 3, using Eq. (1) in the main text (circles). The line shows accepted value of this ratio in the B phase [7, 8]. Statistical error from the fit of the temperature dependence of the frequency shift is smaller than the symbol size. Fits on Supplementary Figs. 2 and 3 give identical results within the symbol size. Uncertainties from the temperature calibration determined as described in Methods and from the finite width of the NMR spectra are both about 1%. We note that this analysis assumes λ_D being independent of pressure, which might be an oversimplification.

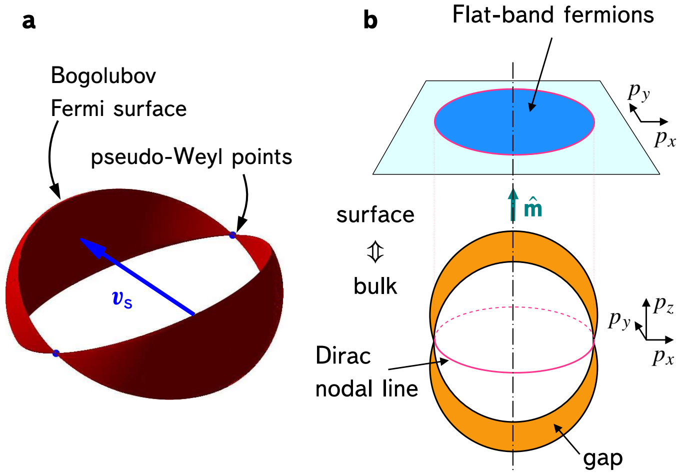

Supplementary Figure 5. Consequences of the nodal line in the polar phase. a, Illustration of the Bogoliubov Fermi surface in the polar phase in the presence of superflow $\mathbf{v}_s$. Under superflow the Dirac nodal line transforms to two Fermi pockets, which touch each other at two points (pseudo-Weyl points) [4]. In the zero-temperature limit, this Fermi surface should lead to the cubic suppression of the gap amplitude as a function of v_s: $1-\Delta(v_s)/\Delta(0) \propto (v_s/v_c)^3$, where $v_c = \Delta(0)/p_F$. **b**, Owing to the surface-bulk correspondence in topological matter with Dirac lines [5, 6], the fermionic flat band appears on the surface normal to the direction of the confining strands.

Supplementary References

[1] Dmitriev, V. V., Senin, A. A., Soldatov, A. A. & Yudin, A. N. Polar phase of superfluid ^{3}He in anisotropic aerogel. *Phys. Rev. Lett.* **115,** 165304 (2015).

[2] Mäkinen, J.T., Dmitriev, V.V., Nissinen, J., Rysti, J., Volovik, G.E., Yudin, A.N., Zhang, K., Eltsov, V.B. Half-quantum vortices and walls bounded by strings in the polar-distorted phases of topological superfluid ^{3}He. *Nat. Comm.* **10,** 237 (2019).

[3] Dmitriev, V.V., Soldatov, A.A. & Yudin, A.N. Superfluid ^{3}He in a nematic aerogel. *JETP* **131**, 2–10 (2020).

[4] Autti, S., Mäkinen, J. T., Rysti, J., Volovik, G. E., Zavjalov, V. V. & Eltsov, V. B. Exceeding the Landau speed limit with topological Bogoliubov Fermi surfaces. *Phys. Rev. Research* **2**, 033013 (2020).

[5] Schnyder, A. P. & Ryu, Sh. Topological phases and surface flat bands in superconductors without inversion symmetry. *Phys. Rev. B* **84,** 060504(R) (2011).

[6] Kopnin, N. B., Heikkilä, T. T. & Volovik, G. E. High-temperature surface superconductivity in topological flat-band systems. *Phys. Rev. B* **83**, 220503(R) (2011).

[7] Serene, J.W & Rainer, D. The quasiclassical approach to superfluid ^{3}He. *Phys. Rep.* **101**, 221–311 (1983).

[8] Greywall, D.S. ^{3}He specific heat and thermometry at millikelvin temperatures. *Phys. Rev. B* **33**, 7520–7538 (1986).

[9] Volovik, G.E. Quantum phase transitions from topology in momentum space, in: *Quantum Analogues: From Phase Transitions to Black Holes and Cosmology*, eds. William G. Unruh and Ralf Schützhold, Springer Lecture Notes in Physics **718** (2007), pp. 31–73.

[10] Hisamitsu, T., Tange, M. & Ikeda, R. Impact of strong anisotropy on the phase diagram of superfluid ^{3}He in aerogels. *Phys. Rev. B* **101,** 100502(R) (2020).
